# Signature Gene Expression Reveals Novel Clues to the Molecular Mechanisms of Dimorphic Transition in *Penicillium marneffei*

**DOI:** 10.1371/journal.pgen.1004662

**Published:** 2014-10-16

**Authors:** Ence Yang, Wang-Ngai Chow, Gang Wang, Patrick C. Y. Woo, Susanna K. P. Lau, Kwok-Yung Yuen, Xiaorong Lin, James J. Cai

**Affiliations:** 1 Department of Veterinary Integrative Biosciences, Texas A&M University, College Station, Texas, United States of America; 2 Department of Microbiology, University of Hong Kong, Hong Kong, China; 3 Department of Biology, Texas A&M University, College Station, Texas, United States of America; MicroTrek Incorporated, United States of America

## Abstract

Systemic dimorphic fungi cause more than one million new infections each year, ranking them among the significant public health challenges currently encountered. *Penicillium marneffei* is a systemic dimorphic fungus endemic to Southeast Asia. The temperature-dependent dimorphic phase transition between mycelium and yeast is considered crucial for the pathogenicity and transmission of *P. marneffei*, but the underlying mechanisms are still poorly understood. Here, we re-sequenced *P. marneffei* strain PM1 using multiple sequencing platforms and assembled the genome using hybrid genome assembly. We determined gene expression levels using RNA sequencing at the mycelial and yeast phases of *P. marneffei*, as well as during phase transition. We classified 2,718 genes with variable expression across conditions into 14 distinct groups, each marked by a signature expression pattern implicated at a certain stage in the dimorphic life cycle. Genes with the same expression patterns tend to be clustered together on the genome, suggesting orchestrated regulations of the transcriptional activities of neighboring genes. Using qRT-PCR, we validated expression levels of all genes in one of clusters highly expressed during the yeast-to-mycelium transition. These included *madsA*, a gene encoding MADS-box transcription factor whose gene family is exclusively expanded in *P. marneffei*. Over-expression of *madsA* drove *P. marneffei* to undergo mycelial growth at 37°C, a condition that restricts the wild-type in the yeast phase. Furthermore, analyses of signature expression patterns suggested diverse roles of secreted proteins at different developmental stages and the potential importance of non-coding RNAs in mycelium-to-yeast transition. We also showed that RNA structural transition in response to temperature changes may be related to the control of thermal dimorphism. Together, our findings have revealed multiple molecular mechanisms that may underlie the dimorphic transition in *P. marneffei*, providing a powerful foundation for identifying molecular targets for mechanism-based interventions.

## Author Summary

*Penicillium marneffei* is a significant dimorphic fungal pathogen capable of causing lethal systemic infections. It grows in a yeast-like form at mammalian body temperature and a mold-like form at ambient temperature. The thermal dimorphism of *P. marneffei* is closely related to its virulence. In the present study, we re-sequenced the genome of *P. marneffei* using Illumina and PacBio sequencing technologies, and simultaneously assembled these newly sequenced reads in different lengths with previously obtained Sanger sequences. This hybrid assembly greatly improved the quality of the genome sequences. Next, we used RNA-seq to measure the global gene expression of *P. marneffei* at different phases and during dimorphic phase transitions. We found that 27% of genes showed signature expression patterns, suggesting that these genes function at different stages in the life cycle of *P. marneffei*. Moreover, genes with same expression patterns tend to be clustered together as neighbors to each other in the genome, suggesting an orchestrated transcriptional regulation for multiple neighboring genes. Over-expression of the MADS-box transcription factor, *madsA*, located in one of these clusters, confirms the function of this gene in driving the yeast-to-mycelia phase transition irrespective of the temperature cues. Our data also implies diverse roles of secreted proteins and non-coding RNAs in dimorphic transition in *P. marneffei*.

## Introduction

Systemic dimorphic fungi are a group of phylogenetically diverse fungal pathogens which are often geographically restricted but pose an increasing threat to the general population, particularly for immunosuppressed hosts. When transferring between their inhabited environments and human body, morphologic shifts seem necessary for dimorphic fungi to adapt to new circumstance [1]. The phase transitions are regulated by temperature in systemic dimorphic fungi, which take the saprotrophic mycelial form at the lower ambient temperature and the pathogenic yeast form at the higher host body temperature [2]. The mycelium-to-yeast (M-Y) transition is believed to be critical for the pathogenicity of systemic dimorphic fungi because the yeast form is the *in vivo* cellular form that is capable of evading the host immune system [3], while the yeast-to-mycelium (Y-M) transition is crucial to maintain an environmental reservoir, since these fungi are not directly transmitted between mammalian hosts [4]. Thus, the mechanisms of dimorphism attract great interest within the scientific community [5].

*Penicillium marneffei* is a strictly thermally dimorphic fungus [6], recently renamed *Talaromyces marneffei* [7]. At 25°C, *P. marneffei* grows vegetatively as mycelia and shows typical multinuclear mold morphology. At 37°C, the fungus undergoes the phase transition with concomitant coupling of nuclear and cellular division to form uninucleate, single-celled yeasts. To date, over forty genes have been functionally characterized in *P. marneffei* (see review [8]), yet genetic mechanisms underlying dimorphism remain elusive. Dimorphic development in *P. marneffei* is a complex process controlled by a suite of genetic elements. The recent advent of high-throughput approaches has brought new promise for the utilization of genomic and systematic applications to complement the conventional single-gene approaches to identify factors and processes that contribute to dimorphism [9]–[13].

In the present study, we employed next-generation sequencing technologies to revisit the genome sequences of *P. marneffei*, and systematically identified signature expression changes associated with the dimorphic switch. Specifically, we conducted a hybrid assembly of the *P. marneffei* genome with data derived from three different sequencing technologies. We also utilized RNA-seq to characterize *P. marneffei* transcriptomes at various stages of its life cycle. Using the over-expression experiment, we investigated the function of an important transcription factor and showed that the activation of this transcription factor can induce mycelial growth of *P. marneffei* at 37°C. We provided evidence for the potential roles of secreted proteins, non-coding RNAs, and secondary structural transition of mRNA transcripts in regulating thermal dimorphism in *P. marneffei*.

## Results

### Hybrid assembly of the *P. marneffei* genome

We previously sequenced the genome of *P. marneffei* PM1 strain using Sanger sequencing and obtained 190.3 Mb of shortgun sequences [14]. In the present study, we re-sequenced the genome using Illumina and PacBio sequencing technologies. We obtained 4.12 Gb of Illumina reads and 91.70 Mb of PacBio reads. The length of PacBio reads ranged from 50 to 15,433 bp with an average of 1,885 bp. As a result, we have sequenced the *P. marneffei* genome using all three generations of sequencing technologies. To take full advantage of reads generated by these different technologies, we adapted a hybrid assembly strategy. The first step of the hybrid assembly involves the error correction for PacBio long reads using massive high-throughput short Illumina reads [15]. This step is essential because the high error rate of PacBio reads would otherwise interfere with the overall assembly. The error correction algorithm is implemented in PacBioToCA of Celera Assembler [16]. The length of seed is a key parameter that influences the results of mapping and error correction. To determine the influence of the seed length on the performance of error correction, we compared the error-corrected PacBio reads against the reads from the Sanger assembly. We found that the accuracy of error correction was not sensitive to the length of seed, while the seed length of 12 produced the largest yield of error-corrected PacBio reads (Figure S1). Thus, the seed length of 12 was used for the error correction. The second step of hybrid assembly is to determine the optimal seed length for full assembly and use it to assemble all three types of reads simultaneously. To do so, we performed the full assembly multiple times by setting the length of seed from 16 to 75. We evaluated the assembly results by the N50 scaffold size (Figure S2). We chose the optimal seed length 62 for the full assembly. The final full assembly was performed using Celera Assembler [16]. To illustrate the performance of hybrid assembly, we also assembled the genome using only Sanger reads and Illumina reads, without PacBio reads. Phrap was used to assemble Sanger reads, while ABySS [17] and SOAPdenovo [18] were used to assemble Illumina reads. Indeed, hybrid assembly produced results better than those obtained by the other non-hybrid means of assembly (Table 1). In addition, we adapted a procedure described in [19] to use the paired-end RNA-seq reads (described below) to further improve the assembly.

**Table 1:** Comparison of genome assembly results derived from different sequencing technologies and assembly strategies.

Technology	Assembler	Optimized k-mer or the length of seed	N50 (kb)	Number of Scaffolds	Total Size (Mb)	Longest Scaffold Size (kb)	Average Length of Scaffolds (kb)
Sanger	Phrap	14	24.08	2730	28.92	178.73	10.59
Illumina	ABySS	28	211.24	680	28.50	871.59	41.91
Illumina	SOAPdenovo	57	170.68	599	27.95	657.48	46.65
Sanger, Illumina, & PacBio	Celera Assembler	62	303.25	416	28.52	1003.92	68.56

The newly assembled genome consists of 28.35 Mb of sequences, distributed on 216 scaffolds. The N50 reaches 678.24 kb, which is 3.5 times longer than the draft assembly we previously reported [14].The longest scaffold is 1.28 Mb. To our knowledge, this is the first time that all three generations of sequencing technologies were used in *de novo* genome assembly for a fungal genome.

Using *ab initio* gene prediction, subsequently improved by using expression data, we annotated 9,480 protein-coding genes and 571 non-coding RNA genes (i.e., genomic loci that can be transcribed into mRNA molecules but with minimal protein-coding potential) (Table S1). For these protein-coding genes, we annotated 6,066 by searching the Swiss-Prot database using BLASTP (Table S2), 5,890 with 1,687 gene ontology (GO) terms (Table S3), and 7,358 with 5,340 IPR names (Table S4).

### Patterns of gene expression under various growth conditions

We used RNA-seq to determine the global gene expression of *P. marneffei* grown on PDA media under four experimental treatments: (1) stable growth at 37°C as yeasts (stable yeast, Y), (2) yeasts grown at 37°C transferred to 25°C for 6 hours (yeast-to-mycelium, Y-to-M), (3) mycelia grown at 25°C transferred to 37°C for 6 hours (mycelium-to-yeast, M-to-Y), and (4) stable growth at 25°C as mycelia (stable mycelium, M). For each treatment, two biological replicates were performed. Highly consistent measures between two replicates were obtained for all treatments (Figure S3). Among 10,051 genes, 92.5% were expressed (FPKM>1.0 in at least one condition).

**Figure 1:**
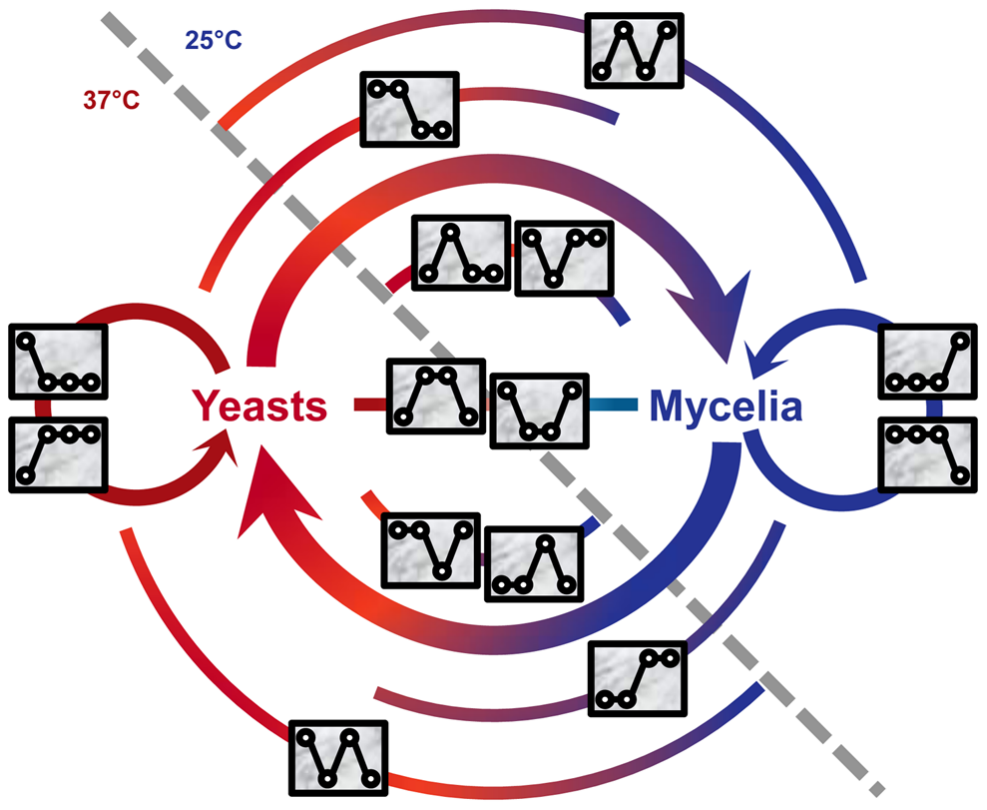
Signature expression patterns and genes implicated in different stages of the life cycle of *P. marneffei*. Two areas divided by the gray dashed line represent the temperatures: 37°C and 25°C, at which *P. marneffei* is grown. Expression patterns, represented with rectangle inserts, are mapped onto the life cycle diagram based on the expression responses of genes and their potential functions. In each rectangle insert, four circles indicate the relative level of gene expressed under the four conditions (i.e., Y, Y-to-M, M-to-Y, and M). Circles are linked by a line to emphasize the difference between signature patterns. The colored arches at the background of the diagram indicate the stages at which genes with corresponding patterns are implicated.

**Figure 2:**
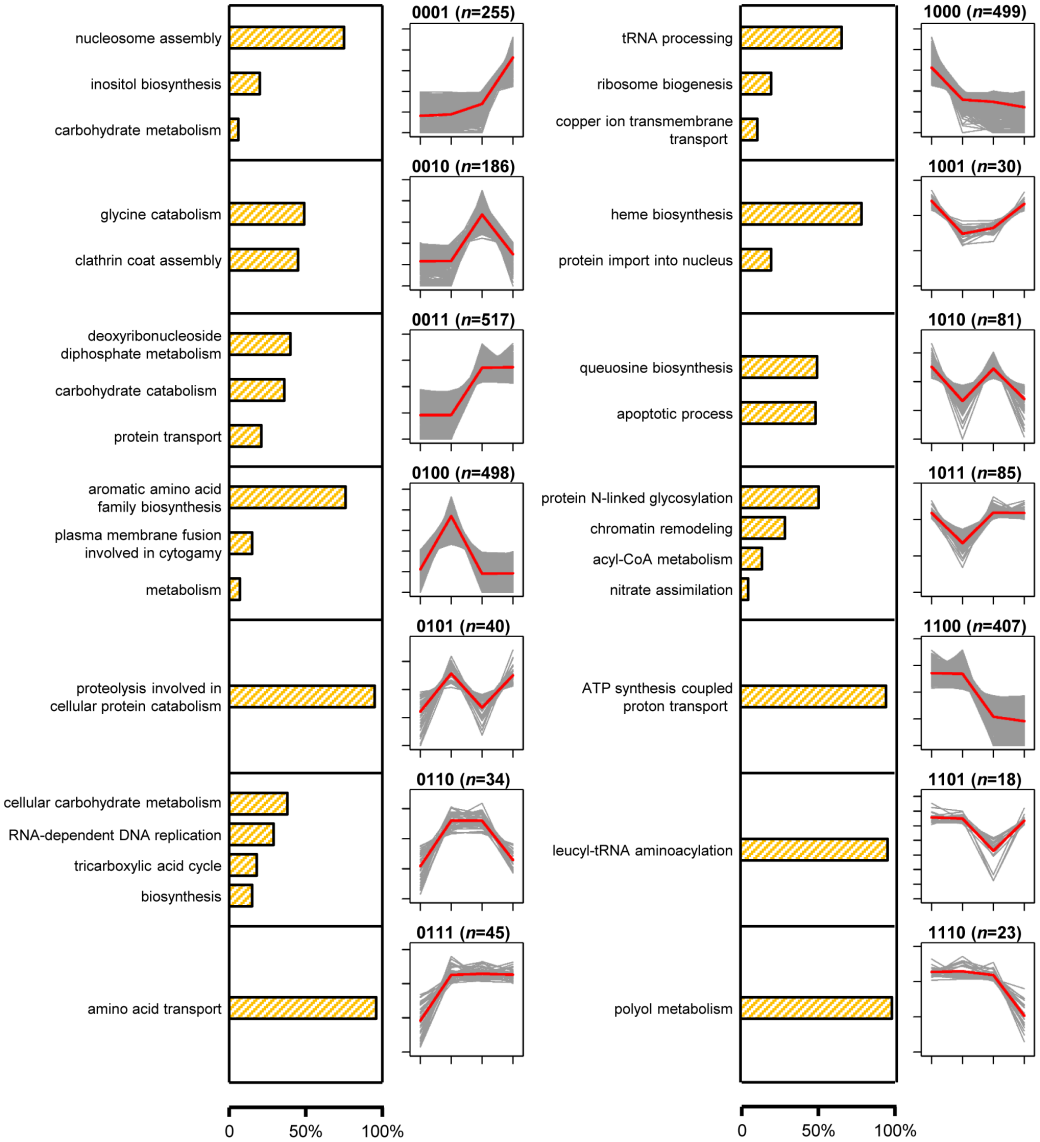
Proportions of genes with the GO term-defined functions and gene expression profiles of 14 signature expression patterns. Bar plots with orange shading show the proportions of genes in each pattern group with functions defined with the summarized GO terms. Line plots show expression profiles of genes in each pattern group. For each gene, normalized expression levels of four conditions: Y, Y-to-M, M-to-Y, and M, are shown with gray line. For each pattern, the average expression levels of four conditions across all genes with the pattern are shown with red line.

We used a four-digit code to denote the expression pattern for each gene. The code is a combination of four “1” or “0”, indicating relatively high or low expression of a gene, respectively, under the four treatments. For example, the expression level of GQ26_0010080 under the second treatment was significantly higher than those of the other three treatment (average FPKM: 182.3, 518.8, 176.8, and 181.9); the gene's expression pattern is “0100”. Note that the expression levels of a gene were compared between treatments of the same gene, not against expression levels of other genes. Genes with the same expression pattern code do not necessarily have the similar overall expression level. This four-digit code system allowed us to create 16 expression patterns and classified all genes into one of pattern groups. The 16 patterns included “0000” for genes that were not expressed (FPKM<1.0) under all four conditions and “1111” for genes expressed under four conditions almost equally. The rest of 14 patterns (such as “0100” and “0011”) were collectively named *signature patterns*. A total of 2,718 *P. marneffei* genes were classified into one of signature pattern groups (Table S1). Each of the 14 signature patterns is presumably implicated at a certain stage in the life cycle of *P. marneffei* (Figure 1). For genes in each pattern group, we tabulated the GO terms associated with gene functions and used REVIGO [20] to summarize information by merging semantically similar GO terms into non-redundant, high-level phrases (Figure 2, Table S5).

### Clusters of genes with same expression patterns

**Figure 3:**
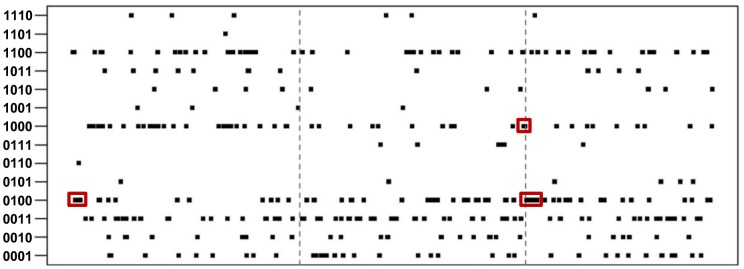
Distribution of genes and signature expression patterns in the top three longest scaffolds. Clusters of genes with the same patterns are highlighted with red boxes.

We examined the distribution of genes with different expression patterns along each scaffold. We found that a number of genes with the same expression patterns form gene clusters (Figure 3). The similarity in expression patterns suggest that the genes sharing the same clusters and thus genetically linked may also play similar or related roles in regulating the life cycle of *P. marneffei*. Of 2,718 genes with 14 signature patterns, 283 (10.4%) are located in 73 clusters (Table 2). These clusters are composed of 3 to 13 genes, scattered all over scaffolds. The size of the clusters (i.e., the number of genes in a cluster) is independent of the type of expression pattern. For example, 23.5% “0100” genes are located within the same clusters. In contrast, 11.4% “0011” and 8.2% “1000” are located in clusters, but the clusters representing each of these patterns have comparable total numbers of genes (498, 517, and 499 for “0100”, “0011” and “1000”, respectively).

**Table 2:** Statistics of expression-pattern clusters and expression patterns.

Pattern	Genes	# of Clusters	Genes in Clusters	# of Gene in Shortest Cluster	# of Genes in Largest Cluster	Average # of Genes in Cluster
1000	499	13	41 (8.2%)	3	5	3.69
0001	255	8	31 (12.2%)	3	7	4.13
0100	498	22	117 (23.5%)	3	13	6.00
0011	517	19	59 (11.4%)	3	5	3.63
0010	186	3	9 (4.8%)	3	3	3.00
0110	34	1	3 (8.8%)	3	3	3.00
1100	407	7	22 (5.4%)	3	4	3.29
All	2718	73	282 (10.4%)			4.34

### MADS-box transcription factors in *P. marneffei*

**Figure 4:**
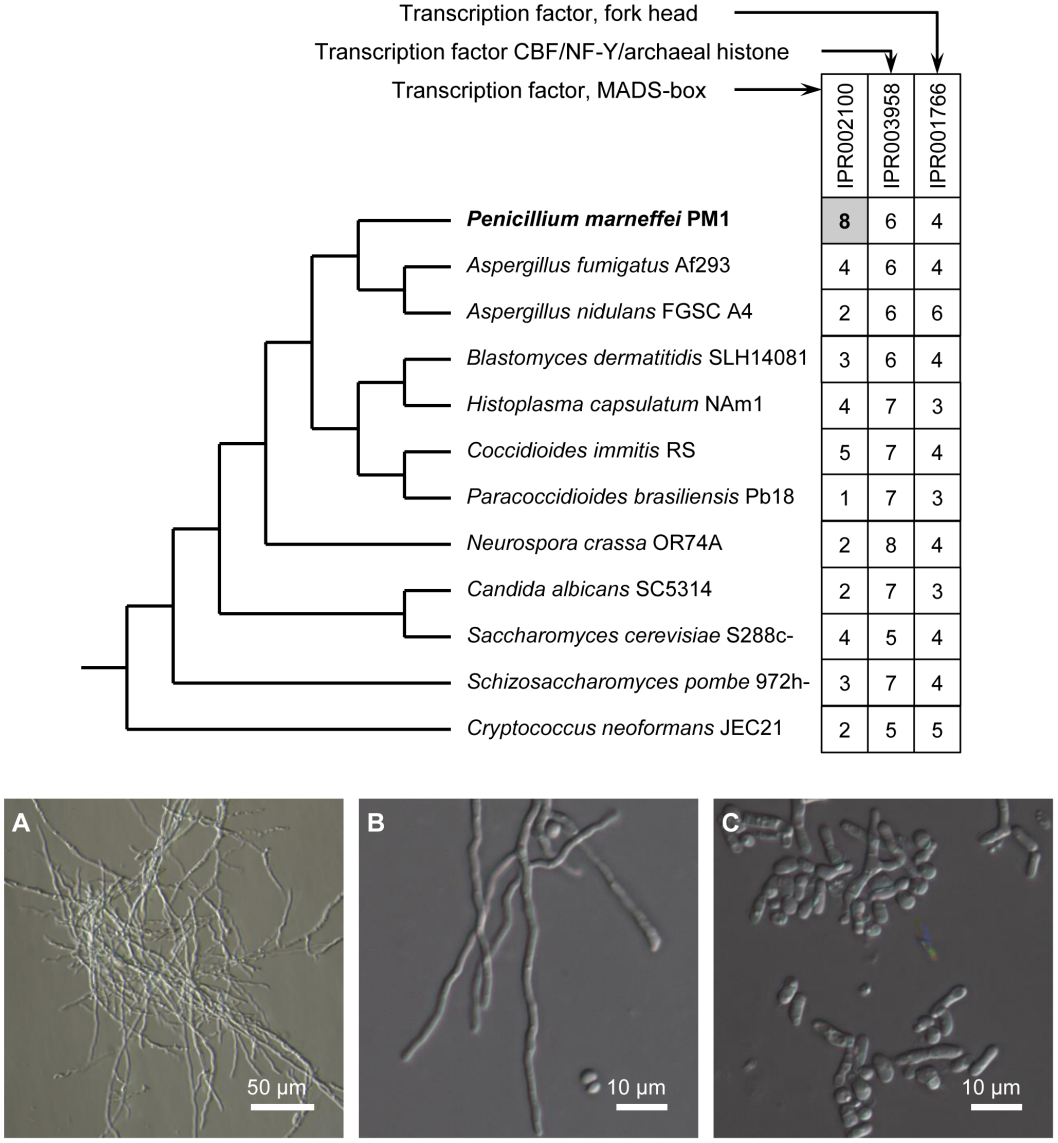
Expansion of MADS-box transcription factor gene family in *P. marneffei* and functional characterization of *madsA*. The upper panel shows the phylogeny of select fungal species and the distribution of three major types of transcription factors. The numbers of genes are given in the boxes. The lower panel shows that overexpression of *madsA* induces mycelia in *P. marneffei* at 37°C. (**A**) A *madsA* ^OE^ mutant strain; (**B**) Another *madsA* ^OE^ mutant strain; (**C**) A wild-type strain of PM1. All strains were pre-cultured on Sabouraud's Dextrose Agar at 37°C for 10 d.

Transcriptional activation or suppression of downstream target genes in response to different stimuli is often accomplished by transcription factors [21], [22]. In *P. marneffei*, genes with three transcription factor domains are the most abundant: *MADS-box* (IPR002100), *CBF/NF-Y/archaeal histone* (IPR003958), and *Fork head* (IPR001766). In particular, the MADS-box transcription factor gene family is clearly expanded in the *P. marneffei* lineage (Figure 4), while the numbers of the other two types of transcription factors are comparable to other fungal species. MADS-box transcription factors are known to regulate cell-type-specific transcription in *Saccharomyces cerevisiae* [23] and *Schizosaccharomyces pombe* [24], [25].

**Figure 5:**
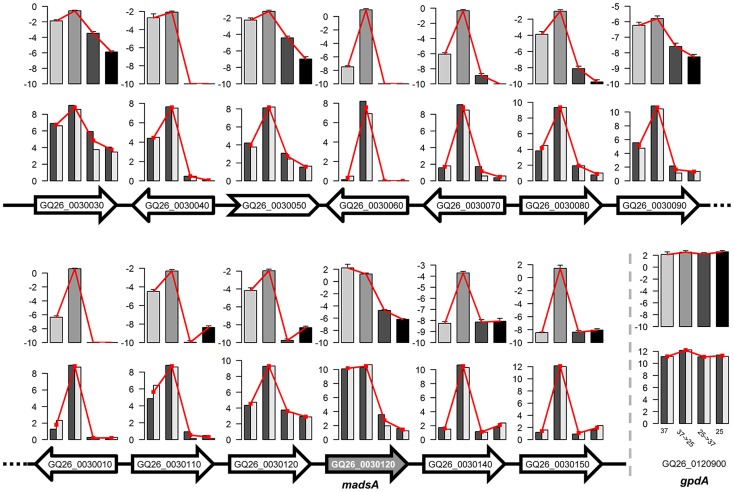
Expression levels of genes in one of expression-pattern clusters. Arrows represent genes and their transcriptional directions on the scaffold. White filled arrows represent yeast-to-mycelium identity genes in the cluster. The gene GQ26_0030130 (i.e., *madsA*) and the non-coding gene GQ26_0030050 are indicted with shading and the arrow of different shape. For each gene, two rows of bar plots show the expression levels of the gene under four treatments measured by two replicates of RNA-seq (bottom) and qRT-PCR (top). For the RNA-seq results, log_2_(FPKM+1) values are shown. For qRT-PCR, the relative gene expression levels (ΔCt to *actA*) are shown. Insert shows the gene expression levels of a house-keeping gene, *gpdA*.

Interestingly, three (out of eight) *P. marneffei* MADS-box transcription factors are separately located in three “0100” clusters (highly expressed in Y-to-M transition). We determined the expression level for genes in one of the clusters using quantitative RT-PCR (qRT-PCR). In this particular cluster, the MADS-box transcription factor (GQ26_0030130) is located in the middle of a group of 12 genes with the expression pattern “0100”. Our qRT-PCR results confirmed that the expression level of all genes in this cluster is significantly up-regulated during Y-M transition (Figure 5). We named the gene GQ26_0030130 *madsA*. In wild-type *P. marneffei*, the expression of *madsA* is up-regulated during Y-M transition, which suggests the role of this gene in stimulating mycelial development. To characterize its function, we overexpressed *madsA* in *P. marneffei* (*madsA* ^OE^) (Materials and Methods). At 25°C, the *madsA* ^OE^ mutant grew as mycelia, showing no morphological differences compared to the wild-type strain. Strikingly, at 37°C, mycelial cells were induced in the *madsA*-overexpressed strain (Figure 4), the wild type cells grew strictly as yeasts at this high temperature. This further supports our hypothesis that MadsA controls the phase transition from yeast to mycelium in *P. marneffei*.

### Secreted proteins in *P. marneffei*

Secreted proteins facilitate the attachment of *P. marneffei* conidia to the bronchoalveolar epithelium of the host [26]. In the newly assembled *P. marneffei* genome, we predicted 434 proteins that are likely to be secreted extracellularly (Materials and Methods). The majority of them (339 or 78.1%) were among those in the 14 signature expression patterns. These predicted secreted proteins appear disproportionally enriched in most of the signature patterns (Table 3). This finding suggests that secreted proteins may play diverse roles at different stages of the *P. marneffei* life cycles. Furthermore, clusters of genes encoding secreted proteins have been identified in non-human pathogenic fungi [27], [28]. For example, 12 clusters containing 79 secreted proteins and ranging from 3 to 26 genes were identified in *Ustilagos maydis* [27], and 121 gene clusters containing 453 secreted proteins and ranging from 3 to 11 genes per cluster in *Monacrosporium haptotylum* [28]. However, in the *P. marneffei* genome, we only found 5 clusters of secreted proteins, each with just 3 genes, suggesting that the clustering organization of secreted proteins *per se* is not important for *P. marneffei* pathogenicity.

**Table 3:** Distributions of secreted proteins and non-coding RNAs in different groups of expression pattern.

Pattern	Description	Genes	Secreted Proteins*	Non-coding RNAs*
0101	25°C sensitive	40	10 (9.3×10^−8^)	0
1010	37°C sensitive	81	15 (6.4×10^−10^)	16 (1.7×10^−6^)
0100	Y-M transition specific	498	71 (<1.0×10^−16^)	14
1011	Y-M transition repressed	85	3	0
0010	M-Y transition specific	186	25 (2.2×10^−11^)	49 (<1.0×10^−16^)
1101	M-Y transition repressed	18	1	0
0110	Transition expressed	34	6 (6.4×10^−4^)	2
1001	Transition repressed	30	2	0
0011	Mycelium and M-Y transition	517	58 (7.7×10^−11^)	15
1100	Yeast and Y-M transition	407	45 (8.4×10^−10^)	37 (0.007)
0001	Mycelium specific	255	43 (1.1×10^−16^)	6
1110	Mycelium repressed	23	7 (1.4×10^−9^)	7 (1.5×10^−5^)
1000	Yeast specific	499	43 (1.4×10^−5^)	37
0111	Yeast repressed	45	10 (7.5×10^−7^)	0

*P-value in parentheses showing significant enrichment (χ^2^ test).

### Temperature-dependent RNA structural transitions in *P. marneffei*

In a previous study, we found that the expression of most fungal heat-responsive genes in *P. marneffei* are not up-regulated at 37°C [13]. This led us to believe that *P. marneffei* may take a distinct strategy of genetic regulation at the elevated temperature beyond known heat-shock proteins [13]. RNA structure is crucial for gene regulation and function [29]. For example, RNA structures near the start codon of the *URE2* transcript reduced its translation rate in *S. cerevisiae* [30]. Parallel analysis of RNA structures with temperature elevation (PARTE) of *S. cerevisiae* revealed that thermodynamically unstable structures are enriched in ribosome binding sites in the 5′-UTRs of mRNAs [31], which suggested that RNA thermometers can function as an evolutionarily conserved heat shock mechanism in eukaryotes [32].

**Figure 6:**
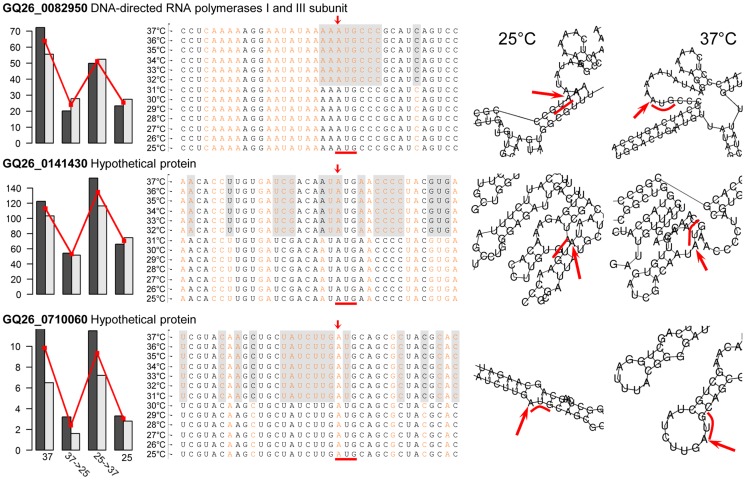
Temperature-induced structural transitions of *P. marneffei* mRNA transcripts. Left panels show the expression levels (in FPKM) of three genes with transcripts whose structures are temperature dependent. Middle panels show mRNA sequences with structural annotation for each nucleotides. Nucleotides in the stem structure are shown in black, those in the loop structure are shown in orange. Nucleotides highlighted in gray shadow are those with structural difference at 37°C versus 25°C. Right panels show the computational predictions of the secondary structures of three mRNAs at 25 and 37°C. For each transcript, the translation initiation site is indicated with red arrow and the start codon AUG is indicted with red bar.

Here we hypothesized that the structural transition of mRNAs at different temperatures is one of the mechanisms underlying thermal dimorphism of *P. marneffei*. To this end, we employed a computational approach based on RNAfold v2.1.7 [33] to determine the secondary structure of *P. marneffei* mRNAs at 25 and 37°C. Through the structural comparison, we identified the mRNAs whose predicted structures are substantially different at the two temperatures. The structural differentiation was assessed by focusing on the region of −9 to +6 base positions around the translation initiation codon. Nucleotides in this region have been shown to be important for the regulation of translation initiation [34]. We expected that this region in mRNAs of temperature-sensitive genes would be more “structurally open” (i.e., contains more unpaired bases) at 37 than at 25°C, facilitating the translation of the mRNAs into proteins. Accordingly, the expression of these genes might also be up-regulated at 37°C. We identified 59 mRNAs structurally more open at 37 than 25°C (Table S6), which was indicated by at least eight more unpaired bases in the translation initiation region at 37°C. Fourteen of these mRNAs are transcribed from genes with one of signature expression patterns (Table S7). Three are transcribed from genes with the expression pattern of “1010”, which indicates that their transcription is highly sensitive to 37°C (Figure 6).

### Non-coding RNA genes in *P. marneffei*

We predicted 571 potential non-coding RNA (ncRNA) genes whose transcripts have no or minimal protein-coding potential, indicated by the lack of significant hits when comparing the transcripts against sequences of the Genbank database using the BLASTX algorithm. The expression patterns of these ncRNAs are more likely to be “0010”, “1010”, “1110”, and “1100” (Table 3). Notably, 8.4% (49 of 571) of the ncRNAs have an expression pattern of “0010”. This figure is significantly higher than the background frequency of 1.9% (i.e., 186 of 10,051 total genes have the pattern of “0010”).

Because ncRNAs are often partially complementary to other molecules and take effect through binding to their targets, we searched all the potential binding sites of the 571 ncRNAs in *P. marneffei* transcripts using the BLASTN-short algorithm. We found a total of 569 genes containing at least one potential ncRNA binding site (Table S8). The expression patterns of these target genes tended to be those related to M-Y transition, including 37°C-sensitive (“1010”), M-Y transition specific (“0010”), and mycelium and M-Y transition (“0011”)(*P* = 5.6×10^−8^, 3.5×10^−5^, and 0.013, respectively; χ^2^ test). Additionally, we found 89 potential binding sites located in the structurally flexible regions as indicated by the differential secondary structure prediction at 25 and 37°C.

## Discussion

Whole-genome sequencing represents a powerful and critical tool for functional genomic studies. Various sequencing technologies have been developed for genome sequencing, but none of them is prefect—all of these technologies have their own advantages and drawbacks. Illumina, for example, delivers high-throughput, inexpensive, and accurate sequence information [35]. However, template amplification is required before sequencing with Illumina, which could cause amplification artifacts [36] and biased coverage [37] related to the chemical-physical properties of the genome. More importantly, Illumina produces short reads, which decreases the continuities of contigs [38]. By contrast, the PacBio sequencer does not require template amplification and thus reduces the composition bias, and is able to produce reads over 10 kb long [39]. These features allow PacBio to bypass the short-read issues of Illumina, and confer the potential advantage to resolve complex repeat regions of the genome. However, the error rate of PacBio reads is as high as 15% [40]. To solve the problem, we used the error correction algorithm to correct as many as possible of the errors in PacBio reads by mapping Illumina reads to them. The Sanger sequencing, representing an earlier generation technology, is still the “gold standard” for validation; however, partially due to the financial considerations, researchers are inclined to use as few Sanger reads as possible. Here, we have set to combine three different generations of sequencing technologies, namely Sanger, Illumina, and PacBio, to produce a better reference *P. marneffei* genome. The hybrid assembly allowed the advantages of different technologies to complement to each other to improve the quality of assembly. There were more reads that could be mapped to the hybrid assembly of PM1 than the previous one assembled with only Sanger reads [14]. Our results showcased the possibility of improving the overall quality of genome assembly through re-sequencing with diverse platforms. Our strategy is a significant improvement in the balance of cost and genome sequence quality.

It is noteworthy that genome sequences of another *P. marneffei* strain ATCC18224 are available in Genbank (Accession ABAR00000000), which are different from those of *P. marneffei* strain PM1 [14]. Our assembly showed that PM1 may have eight chromosomes because telomere tandem repeat sequences were identified at the ends of 16 scaffolds. In contrast, seven chromosomes are present in the ATCC18224 assembly. A karyotype study also suggested seven chromosomes, but, at the same time, the same study estimated that the genome size was between 25.7 to 26.7 Mb [41], which was smaller than the estimates for ATCC18224 and PM1 (∼29.0 Mb for both). It is possible that a chromosome in size of ca. 2 Mb was overlooked by the karyotype study. Nevertheless, more studies are needed to confirm the number of chromosomes of *P. marneffei* and reconcile assemblies from different strains in the future.

Expression levels at four conditions were considered for each gene as part of the expression pattern analysis. This allowed the multiple-way comparisons of gene expression in all conditions and could, in theory, produce a large number of patterns (i.e., all possible combinations of the results of gene expression level comparison between conditions). To simplify the patterns, we employed the “0”–“1” schema to define signature patterns that only capture the essential differences between conditions. As a result, in the experimental data we collected, only 15 patterns (including “1111” representing equally expressed in all conditions) needed to be considered. This significant reduction of the theoretical number of possible expression-level combinations to the number of observed expression patterns in reality suggested a functional constraint in the biological system of *P. marneffei* gene expression. We mapped all expression patterns with associated genes onto the different biological stages in the life cycle of *P. marneffei* (Figure 1). This represents, so far, the most comprehensive expression pattern-based classification of *P. marneffei* genes. One immediate insight we gain based on the classification was the discovery of expression pattern clusters. Genes in the same cluster have the same expression patterns, probably due to coordinated regulation of these genes that have similar or related functions.

The *madsA* gene is in the cluster of high expression during the morphological transition from the yeast to mycelium form. Indeed, over-expression of this MADS-box gene resulted in the morphological change to mycelium form at 37°C that restricts the wild type *P. marneffei* in the yeast form. This MADS-box transcription factor is located in the cluster of a series of genes with the same expression patterns. The induced morphological change validated the function of this gene in regulating morphological development. This result also demonstrated the usefulness of the signature expression patterns and the clusters of genes with the same patterns. This concept was further illustrated by a series of discoveries involving secreted proteins, RNA structural transition, and ncRNAs. First, 78.1% of predicted secreted proteins have expression patterns that are found among the 14 signature patterns. The portion is significantly higher than that of randomly selected genes that have signature expression patterns (*P*<10^−16^, Χ^2^ test). Second, 23.7% (14 out of 59) of mRNAs with a highly differential structure at 25 and 37°C have a signature expression pattern. This suggests a widely-spread impact of the mRNA structural transition at different stages of *P. marneffei* development. Finally, our results describing the expression patterns of ncRNAs and their target genes suggest that ncRNAs may play a role in M-Y transition.

In summary, we have made several steps toward a better understanding of *P. marneffei* thermal dimorphism. Hybrid assembly combined the advantages of different sequencing technologies to improve the quality of genome assembly. Signature expression patterns allowed the prioritization of genes that potentially play important roles in growth regulation and dimorphic development. This strategy was applied to identify the potential master transcription factor, *madsA*, whose function in regulating yeast-to-mycelium transition was then experimentally validated. We anticipate that our overall strategy and approaches can also be used for studying other systemic fungal pathogens.

## Materials and Methods

### Genomic DNA preparation and sequencing

Sanger sequencing of *P. marneffei* strain PM1 was conducted in our previous study [14]. Briefly, genomic DNA was prepared from the arthroconidia of PM1 grown at 37°C as described [42]. Two genomic DNA libraries were made in pUC18 carrying insert sizes from 2.0–3.0 kb and 7.5–8.0 kb, respectively. DNA inserts were prepared by physical shearing using the sonication method [43]. A total of 190.3 Mb of sequence data, which is equivalent to ∼6.6× coverage of the genome, were generated by random shotgun Sanger sequencing. The draft genome sequences were assembled using Phrap (http://www.phrap.org/).

In the present study, the *P. marneffei* strain PM1 genome was re-sequenced using Illumina and PacBio sequencing technologies. The strain was cultured in Sabouraud's Dextrose Agar (SDA) at 25°C for 7 days. Sporulating colonies were covered by 1 ml of sterile phosphate buffed saline (PBS) containing 0.05% (v/v) TWEEN-20. The resulting mixture of conidia and hyphal fragments was withdrawn and filtered by Miracloth. The density of conidia in the filtrate was adjusted to 1×10^8^ conidia/ml. 100 µl of adjusted conidial suspension was inoculated into 50 ml of Sabouraud's Dextrose Broth (SDB). After culturing at 37°C for 7 days, 5 ml of culture was transferred into another 45 ml of SDB and cultured at 37°C for 16 hours. Cells were collected by centrifugation at 3000×g for 5 min. Genomic DNA was extracted using E.Z.N.A. Fungal DNA Kit (Omega Bio-Tek Inc.), following the manufacturer's instructions with a slight modification: RNA was digested by RNase I instead of RNase A. High-throughput Illumina sequencing was performed by the Beijing Genomics Institute (BGI) Americas. Extracted genomic DNA was fragmented randomly and then DNA fragments around 500 bp in size were retained by electrophoresis. Adapters were ligated to DNA fragments. After cluster preparation, 90-bp pair-end sequencing was conducted. This resulted in raw data of 22,861,112 pairs of 90 bp pair-end reads. The reads were trimmed using fastx-trimmer version 0.0.13, based on the base-calling quality reported by FastQC v0.10.1. PacBio sequencing was conducted by the Biomedical Genomics Microarray Facility at University of California, San Diego. The template library, with 10-kb insert was prepared according to the manufacturer's specifications. The sequencing was carried out on a PacBio RS platform following the standard protocol with a C2 sequencing kit at the 1×120-min acquisition mode. The run was carried out with diffusion-based loading and analyzed using the standard primary data analysis. Finally, the SMRT cell of PacBio system yielded 91.70 Mb of sequences from a total of 48,645 continuous long reads.

### Genome assembly

We employed Phrap v1.090518 or the assembly of Sanger reads alone using various values of -minmatch ranging from 14 (default value) to 30. The default value provides the best assembly with a total length of 28.92 Mb and a scaffold N50 of 24.08 kb after removing contigs shorter than 500 bp. Two software tools were used independently for assembling Illumina reads. For ABySS v1.3.7 [17], different values for parameter *k*-mer size (-k) were used, ranging from 13 to 79. A run with a minimum-required contig size of 100 bp and a k-mer length of 28 nt resulted in an assembly with a total length of 28.50 Mbp and a scaffold N50 of 211.24 kbp after removing scaffolds less than 500 bp. For SOAPdenovo v2.01 [18], different values for parameter k-mer size (-K) were used with odd numbers of 13 to 79. A run with a *k*-mer length of 57 nt resulted in an assembly with a total length of 27.95 Mbp and a scaffold N50 of 170.68 kbp after removing scaffolds less than 500 bp. Error correction of PacBio RS reads were implemented by PacBioToCA of Celera Assembler version 7.0 [16] using Illumina reads [15] with seed length (-MerSize) ranging from 10 to 20. Corrected reads were aligned to the contigs assembled with Sanger reads by BLASTN to estimate the accuracy of error correction. Because seed length affected amount of corrected reads but not the identity, the error correction of PacBio reads was performed with a seed length of 12 to produce the largest amount of corrected read outputs. Hybrid *de novo* assembly was performed using Celera Assembler with Sanger, Illumina, and corrected PacBio reads. The different seed lengths ranging from 19 to 79 were tested. A run with a seed length of 62 resulted in the best assembly as indicated by the N50 scaffold size. RNA-seq reads were used to improved scaffolds using customized scripts based on the principle described in a previous study [19].

### Gene prediction and annotation

Protein-coding genes were predicted by using FGENESH (SoftBerry, Mount Kisco, NY) [44] with genome-specific parameters of *Penicillium*. Aided by a total of 107.4 million paired-end RNA-seq reads, 739 additional genes were annotated that had been missed by the *ab initio* prediction. These genes were then searched against the non-redundant protein database of Genbank using BLASTX to measure the coding potential. Together, we annotated a total of 9,480 protein-coding genes and 571 non-coding RNA genes. To further annotate predicted genes, we searched motifs and domains using InterProScan v5.3-46.0 [45] against publicly available databases, including Pfam, PRINTS, PROSTIE, ProDom, SMART, and PANTHER, and then retrieved the GO annotation from the results of InterProScan for all genes.

### RNA preparation and sequencing

At the beginning of the experiment, conidia of strain PM1 were inoculated onto SDA plates and cultured at 25 and 37°C for a week. The germinated cells were transferred onto new SDA plates every week for 2 weeks to establish stable colonies of either the mycelial or yeast growth form. One week before the extraction of total RNA, the homogenous cells were cultured on new SDA plates to obtain fresh cells. For temperature switch experiments, yeast or mycelial growth plates were transferred to 25 or 37°C for 6 hours, respectively. The total RNAs were extracted from each condition for two independent biological replicates using the E.Z.N.A. fungal RNA kit (Omega Bio-Tek), following the manufacturer's instructions with DNase I digestion to eliminate genomic DNA. We adjusted the total RNA concentration according to the DNA content before the standard poly(A)+ RNA-seq was performed. RNA was quantified using Qubit 2.0 fluorometer (Life Technologies, Grand Island, NY). Finally, we obtained ∼13 million 90-bp paired-end reads for each sample. The reads were trimmed using fastx-trimmer based on the quality reported by FastQC.

### Gene expression analysis

RNA-seq short reads were mapped to the annotated genomes using Tophat v2.0.11 [46]. For each sample, ∼2.0 Gb of reads was mapped, representing ∼100× coverage of *P. marneffei* transcriptome. Using SAMMate [47], the gene expression level was measured in FPKM (fragments per kilobase of exon per million fragments mapped) [48]. For each gene, expression levels associated with each of the four experimental treatments were compared to each other and the relative levels are marked by 1 indicating highly expressed and 0 indicating lowly expressed. Genes that were not expressed at all for the four conditions have a pattern of “0000”, while the house-keeping genes consistently expressed at similar levels under all four conditions have a pattern of “1111”. Besides the two patterns for un-expressed and house-keeping genes, 14 other signature patterns (e.g., “1100” and “0100”) were denoted.

### Quantitative real-time PCR

The *P. marneffei* strain was pre-cultured under the same conditions for preparing the RNA-seq samples. The total RNAs were also extracted using the same procedure described above. The concentrations of total RNA were adjusted to 100 µg/ml. Real-time RT-PCR assays were performed using iTaq Universal SYBR Green One-Step Kit (Bio-Rad Laboratories) with primers shown in Table S9. Template total RNA was reverse transcribed and amplified in a Bio-Rad CFX96 Real-Time PCR Detection System (Bio-Rad Laboratories) in 20-µl reaction mixtures containing 10 µl of iTaq universal SYBR Green reaction mix (2×), 0.25 µl of iScript reverse transcriptase, 2 µl of 100 nM of forward and reverse primers mix, 1 µl of total RNA template, and 6.75 µl of nuclease-free water, at 50°C for 10 min, 95°C for 1 min, followed by 30 cycles of 95°C for 10 s and 58°C for 30 seconds. Melting curves were measured from 65°C to 95°C with 0.5°C of increment. Results from actin (*actA*) were used for normalization. There is no significant difference in the gene expression levels of *actA* in different cell types (conidia, mycelia, and yeast cells) [49]. Glyceraldehyde-3-phosphate dehydrogenase-encoding gene (*gpdA*) was used as a negative control to show that the house-keeping gene was expressed constantly across experimental conditions [50].

### Overexpression of *madsA*

The propagation of plasmid DNA was performed by chemical transformation of *Escherichia coli* TOP10 cells (Invitrogen) according to the manufacturer's instructions. Cells were grown overnight at 37°C on Luria-Bertani broth plates supplemented with 50 µg/ml ampicillin, on a 250 rpm shaking incubator. We selected the promoter of *tefA* (transcription elongation factor alpha) to overexpress *madsA* because *tefA* is a house-keeping gene that highly expressed in all contidions. The *tefA*_(p)_::*madsA*::*tefA*_(t)_ fragment was generated by fusion PCR. The promoter region of *tefA* (PMG0060610) was amplified by PCR with forward primer 5′-GAAGATCTTAAGAATTCACTACCAGCAACC-3′ and reverse primer 5′-GGTTCGGTGCCAGCCATGGTGAAGGTTGTGTGTG-3′. The terminator region was amplified with forward primer 5′-TCCGGCGATTGTGGTGAGTGCACGATCTTGGGTG-3′ and reverse primer 5′-GAAGATCTCTATCGCTTCGCACTGTACATA-3′. The coding region of *madsA* was amplified with forward primer 5′-CACACACAACCTTCACCATGGCTGGCACCGAACC-3′ and reverse primer 5′-CACCCAAGATCGTGCACTCACCACAATCGCCGGA-3′. The fusion PCR product was then ligated into the plasmid pAN7-1 [51] at the *Bgl*II restriction site, resulting in plasmid pAN701-1. The resultant plasmid was linearized with *Ahd*I and transformed to wildtype strain PM1 according to previous publications [52], [53]. SDA supplemented with 200 µg/ml hygromycin B was used as selection medium.

### Prediction of secreted proteins

Multiple software tools were jointly used to predict the secreted proteins [54]. WoLF PSORT (http://www.genscript.com/psort/wolf_psort.html [55], SignalP 4.1 (http://www.cbs.dtu.dk/services/SignalP/) [56], and Phobius (http://phobius.sbc.su.se/) [57] were employed to identify signal peptide signatures. The default parameters were used for all the programs. For WoLF PSORT, proteins were considered to be expressed if their extracellular score was larger than 17 [58]. Proteins predicted to be signal-peptide positive by all three programs were taken through two additional filtering steps. First, we excluded possible membrane proteins using TMHMM v2.0 (http://www.cbs.dtu.dk/services/TMHMM/) [59]—a protein was considered to be membrane protein if its transmembrane domains were not located in the first 70 amino acids of the N-terminus [54]. Second, we removed possible endoplasmic reticulum (ER) targeting proteins, predicted by using PS-Scan (http://prosite.expasy.org/scanprosite/) [60] with Prosite accession PS00014. The remaining proteins were considered secreted proteins and used in the subsequent analyses.

### Data access

All data generated are deposited into the NCBI BioProject database under accession numbers: PRJNA251717 and PRJNA251718. More information can be found online (http://pmarneffei.genomezoo.net).

## Supporting Information

Figure S1 The optimized length of seed for error correction of PacBio RS reads. The error correction of PacBio RS reads is implemented via PacBioToCA of Celera Assembler with Illumina short reads. To measure the accuracy of error correction, the error corrected PacBio reads are compared to contigs assembled by independent Sanger reads with Phrap. Our results show that seed length not impact on the accuracy (**A**) and seed length = 12 produces the largest yield of reads (**B**).
(TIF)

Figure S2 The optimized length of seed for hybrid assembly.
(TIF)

Figure S3 Comparison of gene expression levels between biological replicates. Scatter plots compare the gene expression levels, measured by log_2_(FPKM), in the two replicates of each condition. The two axis in all plots represent gene expression level measured by log_2_(FPKM). Non-expressed genes (FPKM<1) in either replicate are removed. Pearson correlation coefficient (*r*) is indicated in each subplot. Red lines represent the theoretical line y = x. Blue lines represent the linear regression of the real data. The linearity of the points suggests that the correlation between two replicates.
(TIF)

Table S1 Gene expression levels of predicted genes.
(XLSX)

Table S2 Protein annotated by searching the Swiss-Prot data base using BLASTP with e-value<1e-10.
(XLSX)

Table S3 GO term map of predicted proteins of *P. marneffei*.
(XLSX)

Table S4 Map of protein domains of *P. marneffei*.
(XLSX)

Table S5 Clustered enriched GO terms in gene expression patterns.
(XLSX)

Table S6 Genes with temperature sensitive translation initial region at 37°C.
(XLSX)

Table S7 Enrichment analysis of expression pattern of genes with temperature sensitive translation initial region.
(XLSX)

Table S8 Enrichment analysis of expression pattern of potential ncRNA binding genes.
(XLSX)

Table S9 Primers for real-time RT-PCR.
(XLSX)
